# Various presentations of the olfactory hallucination in two patients with migraine disease: Case report

**DOI:** 10.3389/fneur.2022.992763

**Published:** 2022-11-14

**Authors:** Omeed Partovi, Amir Soheil Tolebeyan

**Affiliations:** ^1^Medical College of Wisconsin, Wauwatosa, WI, United States; ^2^Tufts Medical Center/Tufts University School of Medicine, Boston, MA, United States

**Keywords:** migraine, migraine aura, olfactory hallucination, Phantosmia, headache

## Abstract

**Objectives:**

To report two different presentations of migraine with the olfactory hallucinations. A case with the typical hallucinatory olfactory symptoms preceding migraine headaches and another case with longstanding olfactory hallucinations.

**Background:**

Migraine prevails in about 12% of the general population, with the migraine aura accountable for at least one-third of these cases. The most common aura is the visual aura, followed by the sensory aura, speech, and motor auras. Olfactory hallucinations preceding the headache phase of migraine are rare. To date, the International Classification of Headache Disorders (ICHD) has not recognized them as a subset of migraine aura.

**Results:**

This report presents a patient with a typical Phantosmia (PO) aura before her migraine headache and a patient who experiences a longstanding PO aura.

**Conclusion:**

The olfactory hallucination may present differently in patients with migraine disease. Based on the clinical significance of migraine with olfactory hallucinations, we propose that the ICHD classify this phenomenon as a subtype of aura in the future. However, larger studies are still required to better assess the pathophysiology of this phenomenon.

## Introduction

The average prevalence of migraines in the population is reported to be around 12% (1). Migraines are painful, recurring headaches that have been traditionally classified into consisting of four phases: prodrome, aura, headache, and postdrome phase (2, 3). However, people's experiences do not always fit these criteria. Many patients who have migraines with aura experience migraines without any aura as well (3), and 4% of patients have aura arise without a headache ever following (4). Aura is a transient sensory disturbance that gradually arises before a migraine headache and can recur (2). About 30% of migraines come about with aura, and the most common symptom associated with migraine aura are visual symptoms, which appear in around 90% of patients (2). Less typical symptoms of migraine aura are sensory defects, speech or language difficulty, motor weakness, and brainstem or retinal disturbances (2). These symptoms typically last between 5 and 60 min and can occur alone or in succession with one another (3). Olfactory hallucinations or Phantosmia (PO) refers to the perception of an odor that does not exist in the person's environment. PO is a qualitative smell disorder which is often related to quantitative smell disorders (anosmia, hyposmia) in infectious diseases (rhinosinusitis), tumors, schizophrenia, seizures, depression, migraine, and neurodegenerative diseases, such as Alzheimer's disease or Parkinson's disease (5–7). Although cases of olfactory hallucinations and their association with migraines have been reported since 1895 (8), PO is not currently classified as a type of migraine aura (9). PO is estimated to occur in about 0.1% of adults who experience migraines (6, 9). However, the prevalence is likely higher because patients do not consider olfaction a pivotal part of migraines. Additionally, the majority of case reports that have explained PO as a noted aura for their patients' migraines have always described their hallucination to occur before the migraine headache. In this report, we explain a similar patient who experiences PO aura before her migraine headache, as well as a patient who experiences PO as a long-standing aura that lasts for several days after the headache resolves.

## Case 1

We report a 53-year-old otherwise healthy female with a history of migraine headaches without aura since her early 20s. The patient had her initial consult with headache medicine in July 2017, where she reported to be currently having 12 headache days a month, with pain localized on the left side of the neck and occipital area less commonly as well. She rates the pain on average as a 4–9/10. After a headache arises, the patient is usually pain-free 2 h after taking over-the-counter pain medication (Acetaminophen/Aspirin/Caffeine) and resting. She reports that her headaches used to arise around the time of her menses and have been easy to control, but the severity and frequency of her headaches have been increasing over the past couple of years. The patient experiences photo and phonophobia with her migraine headaches, but no nausea, vomiting, or dizziness with her headaches.

Interestingly, the patient notes that she can smell cigarette smoke from the beginning of her headaches up to 3–7 days after the headaches are gone. She reports that this olfactory hallucination started several years ago and that the smell of smoke is associated with about 30–50% of her headaches. The cigarette smoke smell persisted with her migraines even when she lost her ability to smell in October of 2020 due to COVID-19. Her PO did not get any better or worse during the time she had COVID-19. After starting Topiramate for prophylaxis (August 2021), her headaches have become more well controlled and less severe in pain with only 3–4 headache days a month. She also reports that her PO has gone away with her headaches since October 2021. The patient has no history of seizures, no family history of migraines, and an unremarkable brain MRI.

## Case 2

We report a 48-year-old female with a history of migraine without aura, anxiety and depression, and vitamin D deficiency presenting for headache management. The patient's headaches began in her teenage years and started to progress in her mid 30s. The patient describes her headaches as a pressure feeling in the occipital or frontal area lasting up to 3 days and enforces the sensation of a sharp pain on the right or left frontal area lasting a few hours. She has about 15 headache days a month and had about 2–3 headaches a month until a few months ago. She has photo/phono phobia, lightheadedness, and nausea with headache and reports that more of her headaches occur toward the end of the day. The headaches are aggravated by heat/weather changes and alleviated by icepack use. She has a family history of migraine in her mother and maternal grandmother. The patient recently noticed that she started having transient visual disturbances, specifically floaters—a type of visual aura—that can last throughout her headache. Occasionally, the patient will smell burning for about 30 min before her headache. She reports that the burning smell of either cigarette smoke or trash always comes first, followed by visual symptoms and migraine headaches. The patient has no history of seizures, regular menstrual cycles, and has an unremarkable brain MRI.

## Discussion

Aura is a recurring transient sensory disturbance reported in 30% of people with migraines (2). The pathophysiology of how migraines with aura arise has been extensively studied in animal models and is strongly believed to involve cortical spreading depression (CSD) of the neurons and glia in the brain. While CSD has mainly modeled how visual aura in migraines comes about, it is believed that the physiology of CSD plays a similar role in all types of migraine aura (10). Therefore, the treatment strategies for migraines, regardless of their aura, are likely similar. In our two patient cases, the second patient had a visual aura that preceded her migraines. In contrast, the first patient did not have any classic presentations of aura with her headaches. However, both patients did experience olfactory hallucinations of cigarette smoke for either some time before or after their migraine headaches. Olfactory hallucinations, involving the smell of cigarette smoke in particular, and their relation to migraines is not a novel discovery. One previous study prospectively followed 11 patients from a tertiary care center who all had olfactory hallucinations that preceded their migraines. One out of the 11 patients imagined the smell of smoke, eight reported a variety of different unpleasant smells (3/8 smelled gas), and two reported hallucinating pleasant smells. The duration of their pre-migraine smells ranged from 3 to 5 min up to 24 h (9). Another case report from 2020 similarly reported a 51-year-old male who experiences olfactory hallucinations of the smell of gas up to 60–180 min before his migraines (10). In comparison, our second patient had a similar reporting of olfactory hallucinations that preceded her migraines for several minutes before. All of these reported patients, including ours, also had no history of seizures or brain abnormalities, revealing that their olfactory hallucinations are likely influenced by the CSD pathophysiology of their migraine. In contrast, our first patient's olfactory hallucinations consist of a longstanding aura that last up to 3–7 days after her migraines. An olfactory hallucination that occurs for several days after the migraine headache resolves has never been reported in the literature. The reason for this difference in symptoms is unknown and warrants further study on how other longstanding migraine auras physiologically persist. Olfactory hallucinations have been traditionally associated with the temporal lobe (11), and it would be interesting to see if the CSD that occur in these types of migraines also has an association with the temporal lobe or rather other cortical areas. Finally, while our patients offer insight into the different ways olfactory hallucinations can present in patients with migraine disease, more investigation on their formal assessment of olfactory function, differences in family/social history and future response to therapeutic interventions needs to be done.

Olfactory hallucinations and their relation to migraine aura have been reported since 1895 (8), and the number of reports about their association continues to increase. The prevalence of olfactory aura is likely underreported (9) because patients and physicians do not know or expect olfactory aura to be a part of migraine prodrome. Based on the historical significance of this phenomenon, we propose that olfactory hallucinations are considered a subtype of migraine aura in the future. However, larger studies are still required to better assess the pathophysiology and classification of this phenomenon.

## Data availability statement

The original contributions presented in the study are included in the article/supplementary material, further inquiries can be directed to the corresponding author/s.

## Ethics statement

Ethical review and approval was not required for the study on human participants in accordance with the local legislation and institutional requirements. The patients/participants provided their written informed consent to participate in this study. Written informed consent was obtained from the individual(s) for the publication of any potentially identifiable images or data included in this article.

## Author contributions

Study conception and design, data collection, and analysis and interpretation of results performed by OP and AT. Manuscript draft preparation performed by OP. All authors have reviewed the manuscript and approve the final version.

## Conflict of interest

The authors declare that the research was conducted in the absence of any commercial or financial relationships that could be construed as a potential conflict of interest.

## Publisher's note

All claims expressed in this article are solely those of the authors and do not necessarily represent those of their affiliated organizations, or those of the publisher, the editors and the reviewers. Any product that may be evaluated in this article, or claim that may be made by its manufacturer, is not guaranteed or endorsed by the publisher.
